# Physicochemical and Mechanical Characterization of Two Self-Curing Composite Resins for Direct Provisional Prostheses

**DOI:** 10.3390/bioengineering12090996

**Published:** 2025-09-18

**Authors:** Oscar Javier Valencia Blanco, Saray Fernández-Hernández, Hector de Llanos-Lanchares, Miquel Punset Fuste, José Angel Delgado García-Menocal, Javier Gil Mur, Aritza Brizuela Velasco

**Affiliations:** 1 Faculty of Health Sciences, European University Miguel de Cervantes, C/Padre Julio Chevalier 2, 47012 Valladolid, Spain; 2 DENS ia Res Grp, Faculty of Health Sciences, European University Miguel de Cervantes, C/Padre Julio Chevalier 2, 47012 Valladolid, Spain; sfernandezh@uemc.es (S.F.-H.); abrizuela@uemc.es (A.B.V.); 3 Department of Prosthodontics and Occlusion, School of Dentistry, University of Oviedo, C/. Catedratico Serrano s/n., 33006 Oviedo, Spain; llanoshector@uniovi.es; 4 Biomaterials, Biomechanics and Tissue Engineering Group (BBT), Universitat Politècnica de Catalunya (UPC), Av. Eduard Maristany, 10–14, 08019 Barcelona, Spain; 5 Bioengineering Institute of Technology, Facultad de Medicina y Ciencias de la Salud, Universidad Internacional de Catalunya, Josep Trueta s/n, Sant Cugat del Vallés, 08195 Barcelona, Spain; jadelgado@uic.es; 6 Bioinspired Oral Biomaterials and Interfaces, Department of Materials Science and Engineering, Universitat Politècnica de Catalunya (UPC), Av. Eduard Maristany 16, 08019 Barcelona, Spain

**Keywords:** Bis-EMA, Bis-GMA, flexural resistance, microhardness, scratch resistance, temporary provisional prosthesis, water absorption, wear rate

## Abstract

In this experimental in vitro study, both the physicochemical and mechanical properties of two self-curing dental composites were compared: Structur 3 (based on Bis-GMA) and Visco III (based on Bis-EMA), which are used for the direct fabrication of temporary dental prostheses. The properties evaluated included flexural strength, toughness, hydrophilicity (measured using the contact angle), density, microhardness, water absorption, and wear and scratch resistance. In terms of flexural strength, Structur 3 exhibited a higher value (127 ± 16 MPa) than Visco III (103 ± 25 MPa). In addition, the dental composite based on Bis-GMA showed a higher toughness (36.52 ± 9.20 mJ) compared to 16.55 ± 7.55 mJ for the dental composite based on Bis-EMA) and a greater displacement to fracture (2.50 ± 0.38 mm compared to 1.72 ± 0.38 mm). However, Visco III showed a higher microhardness (17.045 ± 0.93 HV0.5) compared to Structur 3 (8.10 ± 0.76 HV0.5) and a lower water absorption (11.2 ± 0.4 µg/mm^3^ compared to Structur 3). In wear tests, Structur 3 showed greater wear (0.047 ± 0.021 mm^2^ wear channel area) compared to Visco III (0.031 ± 0.013 mm^2^). Density analysis showed that Visco III is denser (1.5917 ± 0.006 g/cm^3^) than Structur 3 (1.324 ± 0.005 g/cm^3^). Fractography analysis showed that both dental composites exhibited brittle fractures. Contact angle tests revealed a similar hydrophilicity of both dental composites with values below 90°. These differences in properties may be influenced by the filler composition of the two dental composites, as Visco III contains macro-fillers with elements such as aluminum and barium, which increase radiopacity. The conclusion is that Visco III is preferable in terms of durability and resistance, while Structur 3 is more suitable for applications that require flexibility, such as in provisional prostheses with pontics or in situations that require high esthetic quality.

## 1. Introduction

Provisional prostheses (PPs) are essential in restorative dentistry to maintain function and esthetics during the fabrication of the final restoration. Their role is particularly important in implant-supported treatments in the anterior region, where immediate esthetic requirements often precede full osseointegration [1]. Beyond aesthetics, these provisional restorations must replicate the functional characteristics of the definitive prostheses as closely as possible and support the healing of the periodontal or peri-implant tissues by serving as a guide for the surrounding structures [2,3,4]. They are also a valuable diagnostic tool, allowing the assessment and adjustment of occlusal, functional, and aesthetic parameters [4,5] and helping to refine contour, contact points, occlusion [6], and vertical dimension [3]. Furthermore, they serve as a communication tool to harmonize clinical results with the patient’s expectations and provide emotional reassurance. While their retention time in the mouth is sometimes dictated by the time required for final prosthesis fabrication, they are often retained for longer periods of time when used to stabilize occlusion or guide esthetic planning.

The marketed dental composites for the creation of provisional prostheses consist of an organic matrix composed of a mixture of various types of dimethacrylate dental composite monomers, an inorganic filler generally consisting of silica particles of various sizes (nanofillers, microfillers, and macrofillers, or a combination of the latter two, called “hybrids”), and additionally, polymerization reaction initiators, silanizing agents helping bond the organic matrix with the silica, colorants, and reaction initiators that can be photoinitiators (Camphorquinones) or self-initiators (peroxides) [7].

The inorganic fillers, which are generally glass (silicon dioxide), are reinforcing elements of the polymeric structure, giving greater mechanical strength. The size of the particles, their morphology, and their percentage will be very important factors for the mechanical properties of the dental composite, its flexural strength, microhardness, wear resistance, and scratching behavior [7].

The most important dental dimethacrylate monomers are:

- 2,2-bis-[4-(2-hydroxy-3-methacryloxypropoxy) phenyl] propane (Bis-GMA)
- Bisphenol A glycerolate dimethacrylate), bisphenol A ethoxylate dimethacrylate (Bis-EMA)
- 1,6-bis-bis-(methacryloxy-2-ethoxyamino)-2,4,4-trimethylhexane, urethane dimethacrylate monomer (UDMA)
- Triethylene glycol dimethacrylate (TEGDMA)

The most widely used monomer in dentistry is Bis-GMA, patented by Bowen in 1962 [8]. It was the first dental dimethacrylate dental composite. Its high molecular weight (511 r/mol) and low double bond contraction (3.90 mol/kg) provide low volatility, low polymerization shrinkage, fast curing, and rigidity. The high viscosity of Bis-GMA (1200 Pa·s), largely produced by the pendant hydroxyl group, limits the degree of conversion (34.5%) and decreases the possibility of incorporating the filler. For this reason, Bis-GMA must be mixed with other dimethacrylates of lower molecular weight and higher viscosity, such as UDMA and TEGMA [9]. The lower the viscosity of the mixture, the higher the degree of conversion [7].

In constrast, Bis-EMA is a hydrophobic analog of Bis-GMA due to the substitution of the pendant hydroxyl group (-CHOH-) with an epoxy group (-CH_2_-CH_2_-O-). The advantages of this compound are its low viscosity (0.9 Pa·s), low water sorption, low polymerization shrinkage, and high conversion degree (75.5%), making it a substitute for Bis-GMA in commercial formulations [10].

The fabrication of provisional prostheses with self-curing materials is usually carried out directly in the mouth but can also be carried out indirectly in cases with patients with sensitivity. Polymerization factors such as temperature and water absorption are very difficult to control in the oral environment. Water absorption is a critical factor for the long-term performance and functional quality of polymeric dental provisional prostheses and must be controlled to optimize the life of the restoration and patient comfort, as the main detrimental effects include mechanical degradation, reduced longevity, esthetic changes, influence on thermal properties, and increased risk of bacterial colonization. The material is in constant contact with surfaces from which it can absorb water: saliva, crevice fluid, blood, or moisture from the impression material. This can lead to size changes and permanent stains [11]. For the reasons mentioned above, the compounds should be as hydrophobic as possible [9]. The temperature difference and the heat exchange coefficient of the different surfaces with which the material is in contact can influence the polymerization rate and the conversion index within the same dental composite. Internal stresses or weaker areas may also develop.

As is well known, the mechanical properties of composite materials depend on the monomers that are going to polymerize, especially on the lengths of the chains, their degree of polymerization, the branches they may have, and the Tg temperatures they may have associated with them. However, in addition to the properties of the organic phase, the inorganic phase also plays a very important role, especially in improving mechanical properties. The shape, size, and nature of the inorganic component, as well as its proportion in the composite, are the factors that modify hardness, flexural strength, modulus of elasticity, etc., which are so important for the clinical application of these composites. For example, for large cantilever structures or for edentulous pontics, we will need materials with high mechanical properties in terms of flexural strength. These properties will also be important in dental aesthetics, as harder materials allow for much higher quality polishing, which is why they are suitable for the anterior sectors of the mouth, where aesthetics play a much more important role than in the posterior sectors.

The importance of the long-term reliability of prosthetic materials, especially in terms of their mechanical properties, so that they are capable of withstanding the different degrees of mechanical stress to which the structures may be subjected, makes it necessary to study composite material formulations for these applications. On the other hand, it is also necessary to understand composite materials and the possibilities of modifying their chemical compositions to achieve materials that can be polished and improve dental aesthetics. One of the objectives in the field of dental prosthetic materials is to understand the influence of the properties of their constituents in order to optimize their compositions depending on the required purpose [12,13].

The objective of this in vitro experimental study was to compare the physicochemical and mechanical properties of two self-curing composite resins—Visco III (Bis-EMA-based) and Structur 3 (Bis-GMA-based) in order to provide dental clinicians with objective tools that may support decision-making when selecting the most suitable material for the fabrication of provisional fixed prostheses on teeth and implants. The null hypothesis stated that there would be no statistically significant differences between the two materials in any of the evaluated physicochemical or mechanical properties.

## 2. Materials and Methods

**Figure 1:**
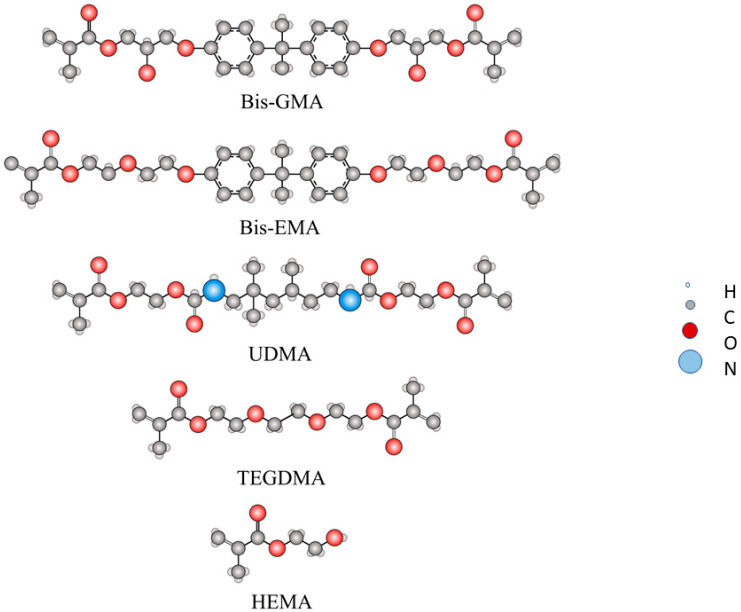
Chemical structures of the components studied.

Two commercially available dental composites for the fabrication of provisional prostheses were evaluated in this in vitro study to compare their performance and properties. These self-curing dental composite samples were presented in specific cartridges for each preparation. The selection of materials was based on their commercial availability and common use in clinical practice. The self-curing dental composite samples were: Structur 3 (VOCO GmbH, Cuxhaven, Germany) for the Bis-GMA-based dental composite and Visco III (Anaxdent GmbHD, Stuttgart, Germany) for the Bis-EMA dental composite. The most important chemical structures for dental components studied are shown in Figure 1. Their respective chemical compositions provided by the manufacturers are presented in Table 1.

The Structure 3 (VOCO) cartridge system with its mixing tips ensures homogeneous mixing and uniform curing of Structure 3, without the need for any type of activation. Once the preparations for printing are complete, the material is applied and inserted into the 316 L austenitic stainless-steel mold to obtain the parts that will be subjected to the various studies. After an intraoral setting time of 45 s, the material remains slightly elastic and can therefore be easily removed from the mold. Four minutes after mixing begins, Structur 3 is sufficiently polymerized to begin finishing the margins and contours. Once curing is complete, simply wipe with a cloth soaked in alcohol to remove the inhibition layer formed by contact with atmospheric oxygen. Finishing with a rotary instrument is not necessary. The preparation of Visco III (Anaxdent) is very similar to that of Structure 3. A Visco III cartridge is placed in the injector of the same company with a small cannula that prevents the formation of air bubbles. The company also guarantees the proper homogenization of the composite. Once injected into the stainless-steel mold, the low-temperature curing process begins, and within 30 s, the material has a setting index that allows the piece to be easily removed from the mold. Three minutes after the start of mixing, Visco III is sufficiently polymerized to begin finishing any excess material or burrs. It is then washed with methyl alcohol to clean any oxidation on the surface. As in the case of Structure 3, it is not necessary to finish with a rotary instrument.

### 2.1. Flexural Strength Determination

For the flexural strength test, (n = 5) specimens of each dental composite with dimensions of 25 × 2 × 2 mm were prepared according to ISO 4049 [14]. The surfaces of the samples were polished with diamond abrasive paper with a 320-grain size (n particles/cm^2^). The materials were stored in water at 37 °C for 24 h. After that time, the flexural strength was determined by the three-point method using a universal testing machine (Zwick/Roell SL, Ulm, Germany) equipped with a 5 kN load cell, a head speed of 0.75 mm·min^−1^, and the distance between the supports was 20 mm. The load was applied at 10 mm from the support [15]. The flexural strength and modulus of elasticity were obtained using the maximum strength value before fracture from the force-elongation curves. The modulus of elasticity was obtained by calculating the slope of the force divided by the cross-sectional area of the sample and the deformation values obtained in the elastic part of the composite material. In all cases, ISO 4049 [14] was followed.

### 2.2. Scanning Electron Microscopy (SEM)

The fracture surfaces of the specimens used in the flexural test were observed in a JEOL JSM 5410 scanning electron microscope (Tokyo, Japan) operated at an acceleration voltage of 10 kV. Prior to observations, the sample surfaces were coated with a gold film to ensure their conductivity using AGB7340 sputtering equipment (Agar scientific Ltd., Stansted, UK) in an argon atmosphere and with a current of 30 mA. For the determination of qualitative chemical composition by energy dispersive X-ray spectroscopy (EDS), the sample surfaces were coated with carbon (C) to avoid overlapping of the silicon (Si).

### 2.3. Surface Roughness

For the quantitative evaluation of surface roughness (n = 5), specimens of each dental composite were prepared as plane-parallel discs (8.5 mm in diameter and 4 mm in thickness) to ensure optimal planarity during the measurements.

A last-generation White Light Interferometry (WLOI) equipment (Optical Profiling System, Wyko NT9300, Veeco Instruments, Tucson, AZ, USA) was used. WLI is a non-contact optical method that allows measurements on 3D structures by using a wave superposition principle with a visible-wavelength light (white light).

Measurements were made using the White Light Optical Interferometry (WLOI) non-contact topography characterization technique with a vertical scanning interferometry mode (VSI). A magnification of 20× with a 1× FOV was used, obtaining an image size of 227 × 298 µm^2^. Three areas were randomly selected on the middle part of the sample surface, and the average of each parameter evaluated was calculated with Wyko Vision 232TM Software (Veeco Instruments, Tucson, AZ, USA).

### 2.4. Contact Angle Determination

For the determination of the contact angle, discs with a diameter of 8.5 mm and a thickness of 4 mm were prepared (n = 7 samples for each dental composite). The number of samples was calculated by power analysis.

The surfaces of the materials were polished with a fine-grain polishing disc 1200 (particles/cm^2^). The samples were washed with distilled water, then with methyl alcohol and acetone. They were dried with hot air flow for total drying. Contact angles were measured using an OCA 11 goniometer (Dataphysics, Filderstadt, Germany). The contact angle was measured once for each specimen using distilled water and a drop volume of 2 µL. The measurements were corrected due to the roughness by the Wenzel equation.

### 2.5. Microhardness Test

Hardness testing was carried out following a methodology adapted from previously published protocols [15]. For each material under investigation, four specimens (n = 4) were fabricated and subsequently evaluated. The number of samples was calculated by power analysis. To calculate the samples hardness, a Vickers indenter with a square-based diamond pyramid tip was used to apply a certain load for a specified time. In this way, after removing the load, hardness can be calculated from the depth or area of the residual impression.

The applied load was HV0.5 (500 g), with a constant load application time of 15 s, using a ×10 magnification objective for subsequent determination of diagonal lengths. A total of 5 indentations were made for each sample to obtain a statistically significant average value. After indentation, the footprint is measured after 15 s of loading. The footprint was determined after 60 and 120 s, and no rebound effect of elastic recovery of the material was observed since the indentation dimensions did not change.

### 2.6. Water Absroption

For the water absorption study of both composite materials, discs with a diameter of 15.0 ± 0.1 mm and a thickness of 1.0 ± 0.1 mm were used.

According to ISO 4049 [14] specifications, a minimum of 3 samples (n = 5 in this study) of discs of each dental composite were placed in a desiccator maintained at 37 ± 2 °C for 22 h, after which they were transferred to a second desiccator maintained at 23 ± 1 °C for 2 h and then weighed with a precision of 0.1 mg [15]. This cycle was repeated until a constant mass was obtained, meaning the mass loss of each sample did not exceed 0.1 mg over a 24-h period.

After bringing the samples to a constant mass, the diameter and thickness of each sample were measured with a precision of 0.01 mm to calculate the area in mm^2^ from the diameter and then, using the thickness, calculate the volume, V, in mm^3^.

Subsequently, the specimens were immersed in distilled water at 37 ± 1 °C for 4 days. The specimens were placed vertically with a separation of 3 mm between them. After 7 days, the samples were removed, washed with distilled water, and dried until free of visible moisture. They were weighed 1 min after being removed from the water. After this weighing, the samples were reconditioned to a constant mass again. Water absorption was evaluated following ISO 4049 guidelines [14]. The specimens were weighed prior to immersion and again after the testing period, and water uptake was calculated from the change in mass.

### 2.7. Wear Resistance Study

Tribological tests were carried out using pin-on-disc equipment from CSEM (Neuchâtel, Switzerland). The equipment provides the coefficient of friction in real time throughout the test and creates a wear channel on the sample. The wear tests were conducted following the recommendations of the international standard ASTM G99 [16]. The wear rate was determined by measuring the area of the wear channel using a Taylor-Hobson profilometer (Leicester, England).

The test conditions were as follows: Applied load of 10 N, linear speed of 10 cm·s^−1^, total test distance of 500 m, Steel spherical pin with a diameter of 6 mm, rotation radius of 4 mm, and 1 test per sample for a total of (n = 3) samples per material. Tests were conducted with lubrication by immersing the samples in Hank’s physiological medium.

### 2.8. Scratch Resistance Evaluation

To determine the scratch resistance behavior of both materials under study, scratch tests were performed using a Revetest—RST equipment from CSM Instruments (Neuchâtel, Switzerland).

The indenter used for scratching the samples was a Rockwell C type, with a conical geometry and a diamond tip. The applied load was 10 N constantly along a constant path of 3 mm in length on the sample surface.

All scratch tests were preceded by a pre-scan at 1 N load along the same path to eliminate the effect of shape and/or possible inclination of the sample in the final measurement.

A total of (n = 3) indentations were made for each evaluated material (dental composite), with one scratch per sample to obtain a statistically significant average value. The study of the results was divided into two parts:

(a) Study of the impression made by the indenter using optical microscopy: from the impression recorded on the sample surface, the width is measured using image processing.
(b) The determination of the scratch channel width was carried out using optical microscopy techniques and subsequent image analysis of the acquired micrographs. For image capture, the Olympus GX51 camera (Tokyo, Japan) with a “JVC F-1030” digital camera and OmniMet Enterprise analysis software (Bühler Technologies GmbH, Ratingen, Germany) was used for this part of the study.

Once the scratch tests were completed, the sample surfaces were coated with a carbon film using PVD-sputtering techniques to improve light reflection and obtain higher-quality and higher-resolution images. The parameters of friction force, dynamic friction coefficient, and indenter penetration depth are evaluated based on the data collected during the test.

### 2.9. Density Determination

The Archimedes method for determining the density of polymeric samples involves first measuring the mass of the sample in air, followed by immersion in a liquid of known density, typically water. The buoyant force acting on the submerged sample produces a measurable reduction in apparent weight, which corresponds to the volume of liquid displaced by the sample. The density of the polymer is then calculated by dividing its mass by the displaced volume, providing a reliable measure of the material’s density. The equipment used for the density measurements was the XS205 Dual Range Balance from Mettler Toledo (Greifensee, Switzerland). For the determination of density, disc-shaped specimens (8.5 mm in diameter and 4 mm in thickness) were prepared (n = 5 per material).

### 2.10. Statistical Study

Statistical analysis was performed using Minitab^®^ version 16.2.1 (Minitab Inc., Reamon, WA, USA). Prior to assessing the normality of the data, Levene’s tests were conducted to evaluate the assumption of homoscedasticity (*p* > 0.05). Prior to conducting inferential tests, normality was assessed for each dataset to determine the appropriate statistical approach. Depending on the distribution, either parametric tests (ANOVA) or non-parametric tests (Kruskal–Wallis) were applied. A 95% confidence interval was used, and differences were considered statistically significant at (*p* < 0.05).

## 3. Results

### 3.1. Flexural Strength

**Figure 2:**
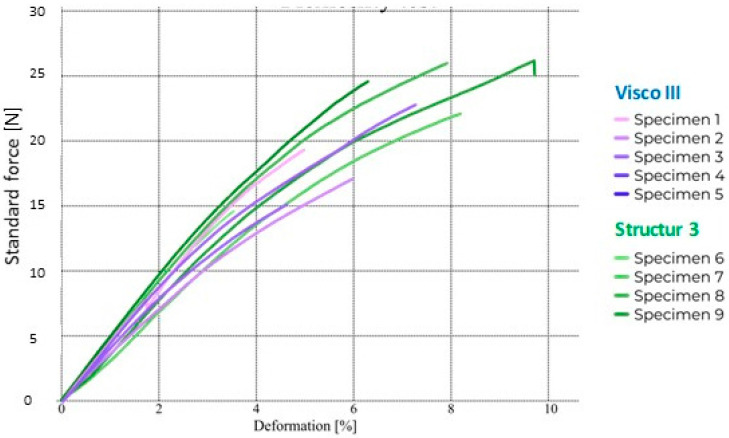
Force-deformation curve of Structur 3 and Visco III dental composites.

The flexural strength results of both dental composites are shown in Table 2. As can be seen, both dental composites exceed the minimum flexural strength value of 80 MPa required by ISO 4049. There are no statistically significant differences (*p* < 0.05) regarding the flexural strength values comparison of both dental composites. In the case of the elastic modulus values, no differences were found between the two dental composites either. Significant differences were found in the values of the area under the stress-strain curve. These results suggest that toughness is higher in the Structur 3 dental composite, meaning more energy is required to fracture this material. Significant differences were also found in the displacement values until fracture, being higher for the Structur 3 dental composite. Figure 2 shows the resulting curves from the mechanical test for both dental composites.

### 3.2. Scanning Electron Microscopy (SEM) Study

#### 3.2.1. Dental Composite Surfaces Study and X-Ray Spectroscopy (EDS)

**Figure 3:**
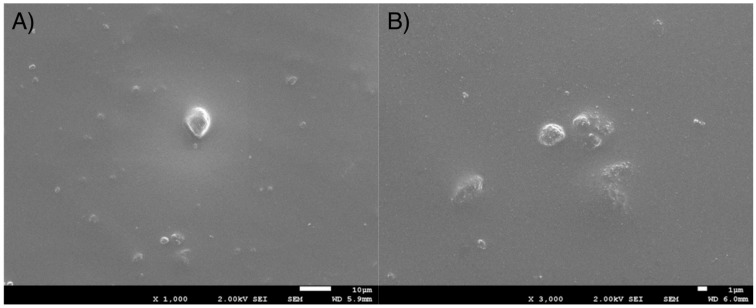
(**A**) Micrograph (1000×) of Structur 3 dental composite Surface, (**B**) Micrograph (3000×) with more detail of Structur 3 dental composite surface.

From Figure 3 can be observed the presence of particles or particle agglomerates of about 2–3 µm in diameter on Structur 3 dental composite surface. However, the average size of the inorganic filler was 0.9 μm of equivalent diameter.

**Figure 4:**
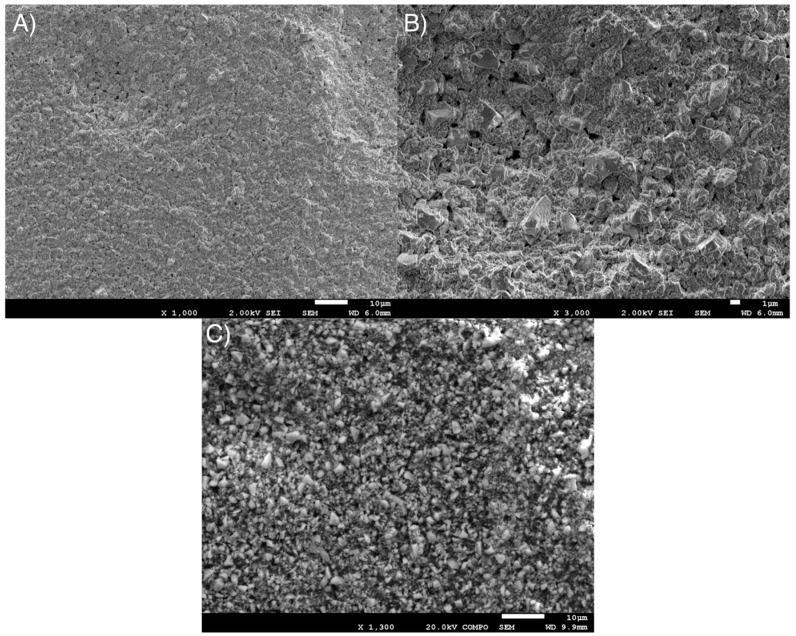
(**A**) Micrograph (1000×) of the Visco III dental composite surface, (**B**,**C**) Micrographs (3000× and 1300×) with more detail of the Visco III dental composite surface.

In Figure 4A–C, details of the surface topography of the Visco III dental composite are shown, which is different in roughness compared to the Structur 3 dental composite. Notably, there is an abundant presence of irregularly shaped particles about 3–4 µm in size.

#### 3.2.2. Fractographic Study by SEM

**Figure 5:**
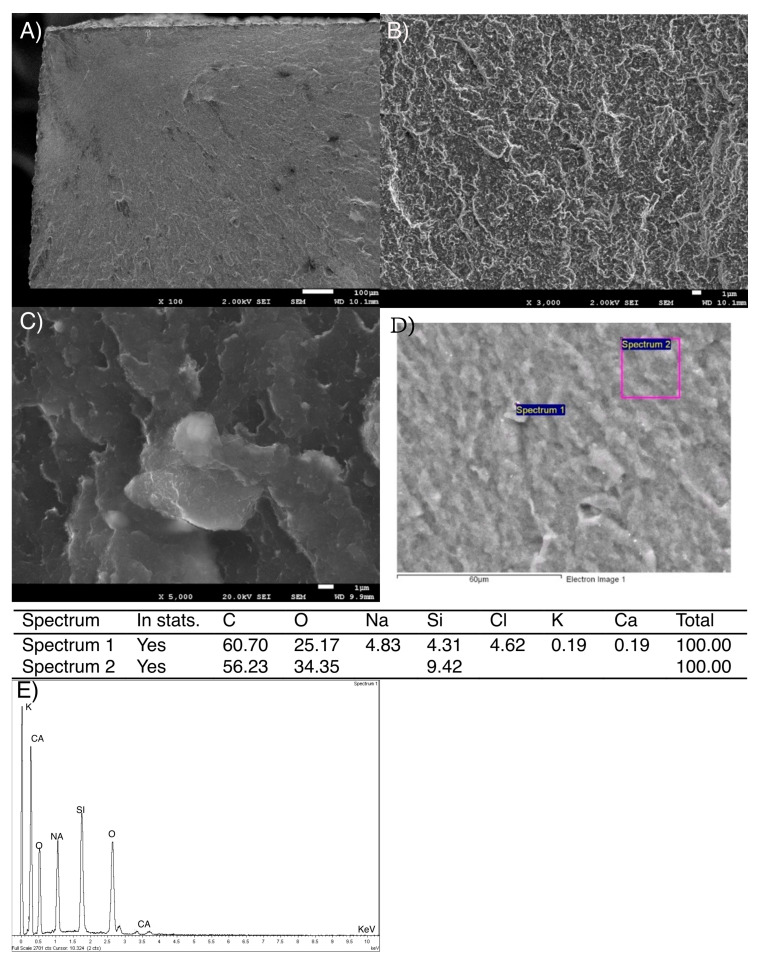
(**A**) Micrograph (100×) of the fracture surface of the Structur 3 dental composite, (**B**) Micrograph (3000×) with more magnification of the fracture surface of the Structur 3 dental composite, (**C**) Micrograph (5000×) of the fracture surface of the Structur 3 dental composite. (**D**). Places of the microanalysis measurements. (**E**). Diffractogram of the Structure 3 realized.

The fracture surfaces of the samples used in the three-point flexural strength test were also studied by SEM. In Figure 5A, the upper left corner shows what could be the area where the brittle fracture of the Structur 3 material began. Figure 5B,C shows details of the rough area of the fracture surface.

In the chemical composition analysis of the fracture surface of the Structur 3 dental composite, the presence of Si corresponding to the inorganic filler of the composite is observed, but the origin of other elements such as chlorine, potassium, sodium, and calcium could not be determined with certainty, although sodium chloride has sometimes been used to regulate the viscosity and swelling of dental composites.

**Figure 6:**
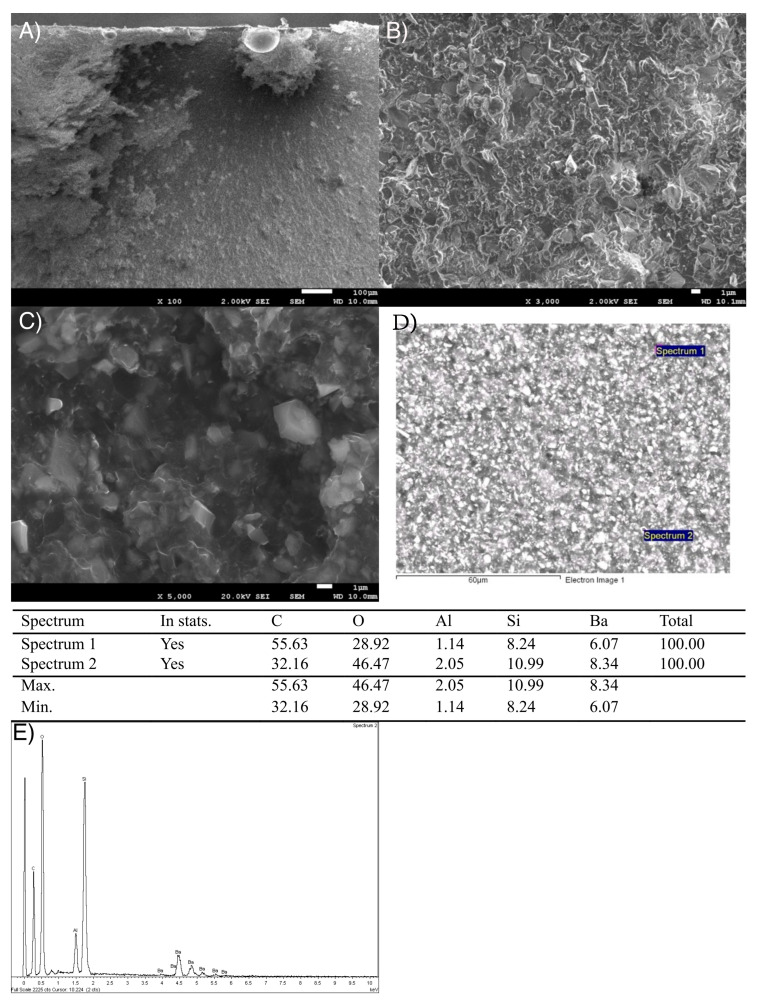
(**A**) Micrograph (100×) of fracture surface in Visco III dental composite. (**B**) Micrograph (3000×) of fracture surface in Visco III dental composite. (**C**) Micrograph (5000×) of fracture surface in Visco III dental composite. (**D**). Places of the microanalysis measurements. (**E**). Diffractogram of the Visco III realized.

The images of the fracture surface of the Visco III dental composite in Figure 6A–C do not clearly show the brittle fracture zone due to the size and quantity of inorganic filler particles, which cause notable surface roughness.

### 3.3. Contact Angle Measurement

Contact angle measurement results are shown in Table 3.

The above results show that there are no differences between the contact angle values for both dental composites, which is related to the similarity in the chemical composition of the polymer matrix of these materials. Contact angle values below 90° suggest that both surfaces are hydrophilic [9].

### 3.4. Density

Table 4 shows the density values obtained for both sample groups. shows the density values obtained for each group of 4 samples (Structur 3 and Visco III), along with the mean values for each group and their standard deviation.

The density values obtained for both materials showed low variability within each group and followed a normal distribution. A statistically significant difference was observed between the two composites (*p* = 0.000), with Visco III exhibiting a higher density than Structur 3.

### 3.5. Microhardness Determination

The HV0.5 hardness values obtained for each group of (n = 4) samples (Structur 3 and Visco III) with a total of 5 indentations per sample are shown in Table 5, along with the mean values for each group and their standard deviation.

The hardness values obtained for Structur 3 and Visco III exhibited low variability within each group and followed a normal distribution. Statistically significant differences were found between the two materials (*p* = 0.000), with Visco III showing higher microhardness values than Structur 3.

### 3.6. Water Absorption

The water absorption values for both dental composites are shown in Table 6.

According to ISO 4049, water absorption values must be ≤40 µg/mm^3^. In this case, both dental composites meet this requirement [14].

### 3.7. Wear Resistance Study

**Figure 7:**
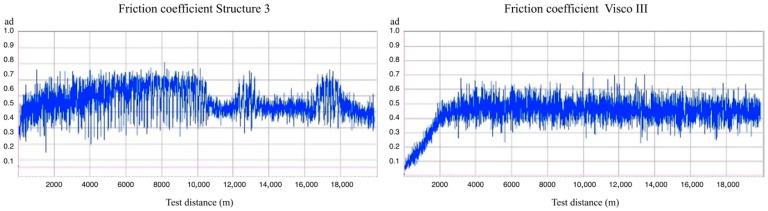
Friction coefficient evolution depending on test distance (m) for both evaluated materials.

Figure 7 shows the representative graphs of the friction coefficient evolution (µ) depending on the distance traveled (d) during the wear test. The comparative analysis between both graphs shows a lower average dynamic friction coefficient value and less fluctuation (variability) of this value in the Structur 3 sample. The results obtained from the wear tests are presented in Table 7, whose preliminary analysis shows a higher wear rate in the Structur 3 material.

The wear rate and wear area data followed a normal distribution. A Student’s *t*-test revealed statistically significant differences between the two composites (*p* = 0.01).

### 3.8. Scratch Resistance Evaluation

**Figure 8:**
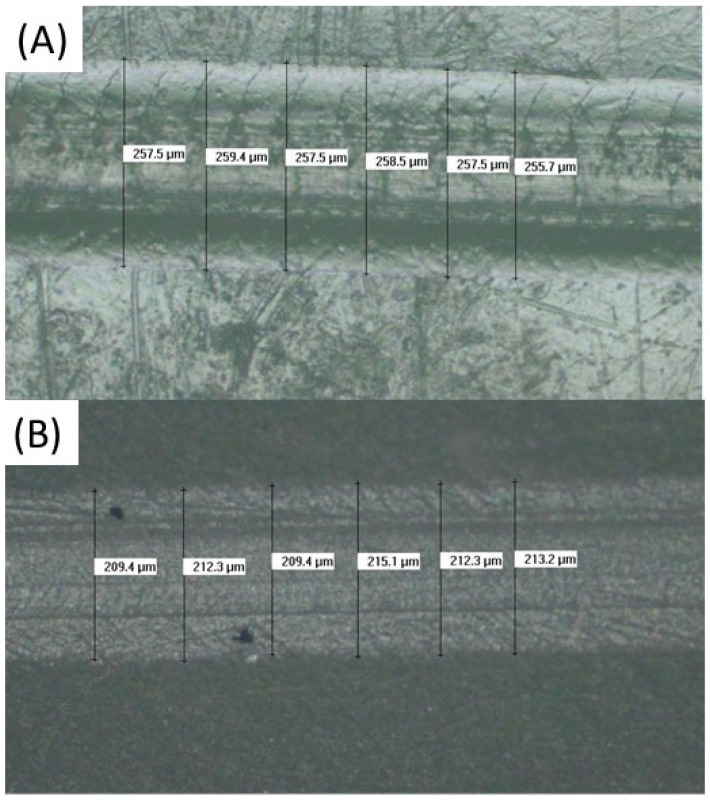
Optical micrographs of scratch marks on both materials: (**A**) Visco III (**B**) Structur 3.

Representative scratch marks for Structur 3 and Visco III are shown in Figure 8. As summarized in Table 8, Visco III exhibited narrower scratch widths (217.0 ± 5.6 µm) compared to Structur 3 (252.4 ± 4.8 µm). The data followed a normal distribution, and a Student’s *t*-test confirmed that the difference was statistically significant (*p* = 0.01). These results indicate higher scratch resistance for Visco III.

**Figure 9:**
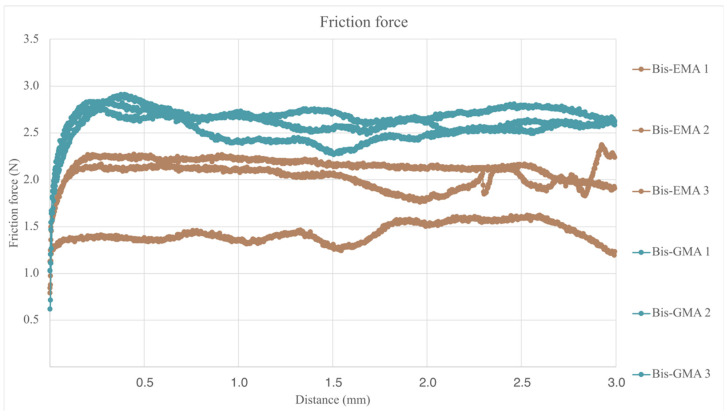
Friction force vs. displacement of both evaluated dental composites.

As can be observed in Figure 9 and in Table 9 results, the samples manufactured in Visco III dental composite reported significantly higher values of friction force during the scratch test.

**Figure 10:**
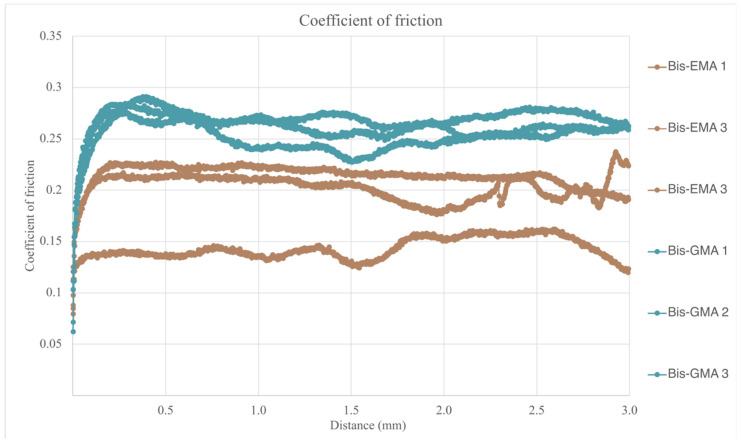
Friction coefficient vs. displacement of both evaluated dental composites.

Once the friction force generated during the test is known, the dynamic friction coefficient can be calculated, taking into account that the applied force was constant and of 10 N, results are summarized in Table 9 and plotted in Figure 10. For the calculation of the dynamic friction coefficient, the values obtained in the central 2.5 mm of the test course were taken into account, excluding the 0.5 mm both at the beginning and at the end of the test course.

The dynamic friction coefficient data did not follow a normal distribution. A Mann–Whitney test confirmed statistically significant differences between the two materials (*p* = 0.000), as illustrated in Figure 10.

**Figure 11:**
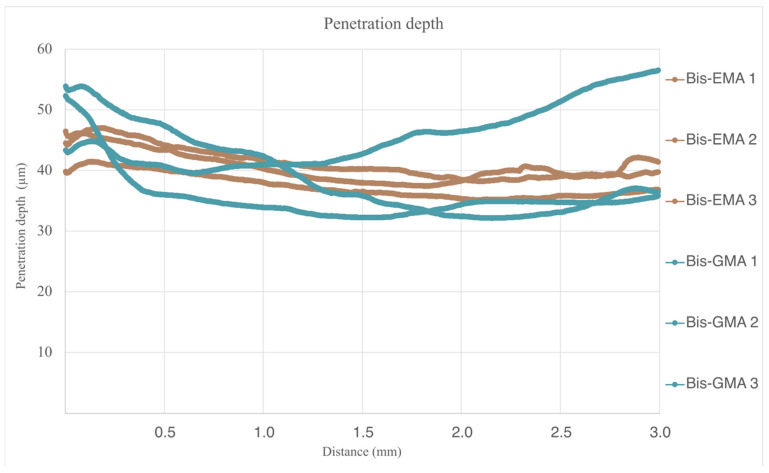
Penetration depth vs. displacement of all evaluated samples.

Figure 11 together with Table 9 shows the penetration depth values of the scratch channel expressed in µm along the scratch test path, whose comparative analysis has allowed us to observe higher and more heterogeneous penetration values in the samples manufactured with the Structur 3 material.

Scratch penetration depth values did not follow a normal distribution. A Mann–Whitney test revealed statistically significant differences between the two materials (*p* = 0.000).

## 4. Discussion

The aim of this in vitro experimental study was to compare the physicochemical and mechanical properties of two self-curing composite resins—Structur 3 (Bis-GMA base) and Visco III (Bis-EMAbase)—for use in provisional prostheses on teeth and implants according to ISO 4049:2019 [14]. Although the test conditions are standardized and controlled, they do not fully represent the oral environment, as they are constantly exposed to saliva, microorganisms, and temperature fluctuations [17]. With the exception of the bending test (which was performed after immersion in water), all other measurements were performed under dry conditions. One limitation of the present study is the lack of aging protocols and liquid media that could more closely replicate clinical conditions. To overcome this, future research will incorporate thermocycling and artificial saliva immersion in order to more reliably simulate the long-term performance of these prosthetic materials within the oral environment. Future studies should simulate intraoral degradation to better assess long-term clinical performance.

Both materials used are commercially available; however, the exact compositions are partly unknown, as manufacturers usually only disclose the components of the material safety data sheet. This limits our ability to correlate specific compositional differences with the observed behavior. Among the limitations of the present study, it is important to consider the potential biocompatibility implications of polymer degradation products, particularly with regard to their possible cytotoxic effects on peri-prosthetic tissues, such as dental bone and gingival structures.

### 4.1. Mechanical Properties

Structur 3 showed a higher flexural strength (127 ± 16 MPa) than Visco III (103 ± 25 MPa), although both have similar moduli of elasticity (1.92 GPa). Gajewski et al. [18], using pure Bis-GMA and Bis-EMA monomers with photoinitiators, reported lower flexural strengths (87.3 ± 16.5 MPa and 72.4 ± 13.6 MPa, respectively) and moduli (1.1 ± 0.3 GPa and 1.0 ± 0.2 GPa, respectively). Our higher values probably reflect the influence of added fillers and comonomers in commercial formulations. The relatively lower strength of Visco III could possibly be improved by adjusting the formulation.

In this sense, the superior mechanical performance of Structur 3 may be attributed to several structural features. SEM analysis revealed a finer, more homogeneous filler distribution than Visco III, where larger, irregular particles were observed. It is known that finer fillers delay crack propagation and thus increase fatigue resistance. In addition, Structur 3 contains UDMA, which has been shown to increase the degree of transformation in Bis-GMA-based systems [19]. Gonçalves et al. [20] demonstrated that UDMA and TEGDMA improve tensile and flexural properties, especially in combination with Bis-GMA. Although Bis-GMA-based composites are generally stiffer due to their bulky, aromatic molecular structure, our results show similar elastic moduli between both materials. This could be due to balancing factors such as crosslink density, filler content, and polymer network architecture.

Park et al. [21] have already found that filler contents below 12% have little effect on the elastic modulus. Our results are consistent with this, as both Structur 3 and Visco III exhibited similar moduli despite different matrix compositions.

On the other hand, Structur 3 also exhibited significantly higher toughness and displacement before fracture. This is evidenced by the larger area under the bending curve and the more pronounced damage to the fracture surface, indicating greater energy absorption. The higher flexibility and smaller filler size likely contribute to these properties, as does the stronger hydrogen bonding potential of the Bis-GMA molecules due to hydroxyl crosslinking [22]. These bonds require more energy to be broken, which may explain the higher toughness of Structur 3.

In contrast, Visco III exhibited higher hardness (17.05 ± 0.93 HV) and density (1.59 g/cm^3^), likely due to the ethoxylated Bis-EMA structure, which allows for tighter molecular packing and more efficient polymer network formation. The higher inorganic filler content further supports this mechanical profile.

Fractographic SEM images confirmed that both materials fracture brittlely, although this is more pronounced in Visco III. This sudden failure mode raises clinical concerns, as the fractures occur without warning and can lead to sharp edges in the mouth. In addition, Visco III exhibited larger, angular filler particles (3–4 µm), which may act as stress concentrators and facilitate cracking. In contrast, Structur 3 had smaller (2–3 µm), rounder particles and a higher measured silica content (14.4 ± 0.96% vs. 12.33 ± 0.76%), partly due to microfillers that are not visible at standard SEM magnifications.

Indeed, the morphology of the particles seems to play a crucial role in the mechanical behavior. The irregular polyhedral shape of Visco III fillers probably contributes to their lower toughness [23]. In contrast, spherical fillers such as those in Structur 3 are associated with smoother surfaces, better polishability, and less wear. Although roughness has no significant effect on the adhesion of Streptococcus mutans or S. mitis [24], smoother surfaces may benefit esthetics and biofilm control.

Hydrogen bonds also influence polymer properties. Studies by Lemon et al. [25] and Barszczewska-Rybarek et al. [26] showed that Bis-GMA has stronger hydrogen bonds compared to UDMA, with intermolecular interactions making a greater contribution. While UDMA-based polymers tend to achieve higher conversion rates, the bond strength of Bis-GMA can compensate for this by improving mechanical performance [9,27].

### 4.2. Physical Chemical Properties

Both materials exhibited similar hydrophobicity, with contact angles of 75.5° (Structur 3) and 76.9° (Visco III), consistent with values above 60° [28]. Although Bis-GMA contains hydroxyl groups, its surface properties—possibly influenced by filler distribution—did not lead to greater hydrophilicity. Studies have not found a consistent relationship between hydrophobicity and bacterial adhesion [29,30], suggesting similar clinical performance in this regard.

Meanwhile, water absorption is a key factor in the degradation of the material. Structur 3 showed higher sorption (15.0 ± 0.7 µg/mm^3^) than Visco III (11.2 ± 0.4 µg/mm^3^). The absorbed water can plasticize the matrix, which can lead to a reduction in hardness and stiffness as well as dimensional changes or pigment infiltration [11,31,32,33]. On the other hand, moderate water absorption can help to compensate for polymerization shrinkage and improve marginal sealing [34,35]. The higher absorption in Structur 3 is consistent with previous findings on Bis-GMA systems and may be related to the lower filler content compared to Visco III.

The higher density of Visco III is consistent with its molecular structure and higher inorganic content. Although Bis-GMA has a stiffer core, the ethoxylated chains in Bis-EMA promote denser packing and denser cured materials [19].

Structur 3 performed better than Visco III in wear and scratch resistance tests, likely due to its greater flexibility and energy absorption capacity. Tougher materials can deform and relieve stress instead of breaking. In addition, water absorption may have a plasticizing effect [36], which improves the wear behavior of the material. Interestingly, harder materials do not always exhibit better wear resistance, as the surface interactions also depend on the morphology and distribution of the fillers as well as the matrix-filler adhesion [19,37].

### 4.3. Surface Properties and Wear Resistance

Moreover, surface roughness also plays a role. Visco III with larger, angular fillers exhibited higher surface roughness, which contributed to greater friction and material loss [38,39,40]. The smoother surface of Structur 3 without visible nanofillers required less force to slide objects and showed better tribological behavior. This factor probably explains its better scratch and wear resistance [41,42,43]. Although Visco III had a higher microhardness, Structur 3 performed better in terms of wear, which supports the assumption that the geometry and flexibility of the fillers are decisive factors.

During wear, composite residues are released, which may degrade in the oral cavity under the influence of water, salivary enzymes, and microbial metabolites such as ethanol [9,27,28,29]. These degradation products may include low molecular weight compounds that pose potential biocompatibility issues [44,45,46,47,48,49].

Structure 3 composite is indicated for temporary prosthetic rehabilitation with intermediate edentulous spaces. In this case, a material with greater mechanical properties is necessary due to the increased stress caused by the lack of teeth. Structure 3 composite is also clinically recommended for anterior spaces, where greater aesthetics are required. As observed in the results, Structure 3 composite is easier to polish and therefore more aesthetic. However, Visco III is indicated for posterior areas where aesthetics do not play an important role and suffers less wear than Structure 3 because it has a higher inorganic filler content, resulting in much less wear.

Future studies should include more clinically relevant aging protocols, such as thermal cycling, exposure to artificial saliva, and bacterial colonization models. It would also be useful to isolate variables such as filler morphology and matrix composition to better understand their individual contributions. While only static flexural tests were performed here, fatigue testing under masticatory loading and physiological conditions is warranted. This characterization of the different composites should be complemented by fatigue studies, i.e., the behavior of mechanical cycles in a physiological environment. In this case, Cosola et al. have reported very interesting work establishing fatigue testing on splinted dental implants [50]. The influence of inorganic filler, its size, nature, and morphology on dynamic mechanical properties in a physiological environment at 37 °C is very important. In principle, it should be expected that materials with greater toughness should have greater fatigue resistance, but this should be demonstrated in future work. Much work has been done on titanium structures and dental implants, as they are homogeneous and highly reproducible materials, although there may be factors that can affect them: roughness, grain size, residual stress... but further work is needed on composite materials for dental use [51,52,53].

In summary, this study provides a comprehensive physicochemical comparison of two widely used composite resins for direct provisional prostheses. The results highlight different mechanical profiles that may support differentiated clinical indications beyond what is commonly claimed by manufacturers. The results of this in vitro study show differences in the physico-chemical behavior of the two dental composites, which may lead to different clinical applications. Accordingly, the null hypothesis, which assumed no statistically significant differences between the evaluated properties of the two materials, must be rejected.

## 5. Conclusions

The Visco III dental composite exhibits several more favorable mechanical properties (higher flexural strength and hardness) than Structur 3. In addition, the polyhedral shape and the large size of the particles reduce toughness. Visco III also has lower water absorption, which could extend its life in the oral environment. On the other hand, Structur 3 has a higher toughness and deformability and a lower wear rate. This dental composite would therefore be indicated for provisionals with pontics (without direct abutment support) to provide greater resistance to flexion when these points are loaded or when a smoother and glossier result is desired, such as in the rehabilitation of esthetic areas. Based on these results, Structur 3 is suitable for long-term temporary treatments or for patients with parafunctional habits such as teeth grinding that lead to wear.

## Figures and Tables

**Table 1:** Dental composite composition provided by manufacturer.

Monomer	Bis-GMA	Bis-EMA
Brand	Structur 3	Visco III
Manufacturer	VOCO GmbH Cuxhaven	Anaxdent GmbH D-Stuttgart Germany
Dental composite type	Self-curing	Self-curing
Composition	Polymers Bis-GMA 5–10% UDMA 10–25% Inorganic Filler Filler (SiO_2_) 0.9 mm 12–17% Amines Terpenes Benzoyl Peroxide Butylated hydroxytoluene	Polymers Bis-EMA 7–12% UDMA 10–25% 1,4-butanediol dimethacrylate (BDDM) 5–7% Filler (SiO_2_) 1.7 mm 17–20% Catalyst Silanizer pyrogenic silicic acid

**Table 2:** Mechanical properties of the studied dental composites (Standard deviations).

Dental Composite	Flexural Strength (MPa)	Modulus of Elasticity (GPa))	Toughness (mJ)	Displacement Until Fracture (mm)
Structur 3	127 (16) ^(^*^)^	1.92 (0.10) ^(^*^)^	36.52 (9.20) ^(^*^)^	2.50 (0.38) ^(^*^)^
Visco III	103 (25) ^(^*^)^	1.92 (0.27) ^(^*^)^	16.55 (7.55) ^(^**^)^	1.72 (0.38) ^(^**^)^

^(^*^), (^**^)^: Different symbols indicate significant differences between materials (*p* < 0.05).

**Table 3:** Contact angle values for both dental composites (Standard deviations).

Dental Composite	Roughness (mm)	Contact Angle, θ
Structur 3	0.74 (0.06) ^(^*^)^	75.8 (2.6) ^(^*^)^
Visco III	0.81 (0.07) ^(^*^)^	77.3 (5.0) ^(^*^)^

^(^*^)^: Different symbols indicate significant differences between materials (*p* < 0.05).

**Table 4:** Density values obtained for both sample groups. (SD:standard deviation).

Material	Structur 3 (g/cm^3^)	Visco III (g/cm^3^)
X	1.324 ^(^*^)^	1.592 ^(^**^)^
SD	0.005	0.006

^(^*^), (^**^)^: Different symbols indicate significant differences between materials (*p* < 0.05).

**Table 5:** Hardness values obtained in both sample groups (Standard deviations).

Material	Hardness (HV)
Structur 3	8.10 (0.76) ^(^*^)^
Visco III	17.05 (0.93) ^(^**^)^

^(^*^), (^**^)^: Different symbols indicate significant differences between materials (*p* < 0.05).

**Table 6:** Water absorption test results (Standard deviations).

Material	Water Absorption (µg/mm^3^)
Structur 3	15.0 (0.7) ^(^*^)^
Visco III	11.2 (0.4) ^(^**^)^

^(^*^), (^**^)^: Different symbols indicate significant differences between materials (*p* < 0.05).

**Table 7:** Pin-on-disc test results of evaluated samples (Standard deviations).

		Material	
Property/Units	Result	Structur 3	Visco III
Wear track area/(mm^2^)	Mean	0.031 (0.013) ^(^*^)^	0.047(0.021) ^(^*^)^
Maximum Friction Coefficient/ad	Mean	0.740 (0.028) ^(^*^)^	0.727(0.071) ^(^*^)^
Average Friction Coefficient/ad	Mean	0.446(0.043) ^(^*^)^	0.416(0.148) ^(^*^)^
Wear Rate/(m^3^/Nm) × 10^−13^	Mean	1.531(0.563) ^(^*^)^	2.342(1.091) ^(^**^)^

^(^*^), (^**^)^: Different symbols indicate significant differences between materials (*p* < 0.05).

**Table 8:** Scratch mark width results (Standard deviations).

Material	Scratch Mark Width, [µm]
Structur 3	252.4 (3.8) ^(^*^)^
Visco III	217.0 (3.4) ^(^**^)^

^(^*^), (^**^)^: Different symbols indicate significant differences between materials (*p* < 0.05).

**Table 9:** General summary of friction force results, dynamic friction coefficient, and penetration depth versus displacement in the evaluated simples (Standard deviations).

Material	Friction Force, (N)	Dynamic Friction Coef., [a.d]	Penetration Depth, (µm)
Structur 3	1.878 (0.386) ^(^*^)^	0.259 (0.011) ^(^*^)^	38.98 (1.87) ^(^*^)^
Visco III	2.591 (0.107) ^(^**^)^	0.188 (0.039) ^(^**^)^	38.34 (5.05) ^(^*^)^

^(^*^), (^**^)^: Different symbols indicate significant differences between materials (*p* < 0.05).

## Data Availability

The original contributions presented in this study are included in the paper. Further inquiries can be directed to the corresponding author.
